# Mesenchymal stem cell-derived exosomal microRNA-133b suppresses glioma progression via Wnt/β-catenin signaling pathway by targeting EZH2

**DOI:** 10.1186/s13287-019-1446-z

**Published:** 2019-12-16

**Authors:** Haiyang Xu, Guifang Zhao, Yu Zhang, Hong Jiang, Weiyao Wang, Donghai Zhao, Jin Hong, Hongquan Yu, Ling Qi

**Affiliations:** 1 Department of Oncological Neurosurgery, First Hospital of Jilin University, No. 71, Xinmin Street, Changchun, 130021 Jilin Province People’s Republic of China; 2 The Sixth Affiliated Hospital of Guangzhou Medical University, Qingyuan People’s Hospital, B24, Yinquan South Road, Qingyuan, 511518 Guangdong Province People’s Republic of China; 3 Department of Pathophysiology, Jilin Medical University, No. 5, Jilin Street, Jilin, 132013 Jilin Province People’s Republic of China; 4 Department of Neurovascular, First Hospital of Jilin University, Changchun, 130021 People’s Republic of China; 5 Department of Ophthalmology, China-Japan Union Hospital of Jilin University, Changchun, 130033 People’s Republic of China

**Keywords:** Mesenchymal stem cells, Exosomes, MicroRNA-133b, Glioma, EZH2, Wnt/β-catenin signaling pathway

## Abstract

**Background:**

Mesenchymal stem cells (MSCs) play a significant role in cancer initiation and metastasis, sometimes by releasing exosomes that mediate cell communication by delivering microRNAs (miRNAs). This study aimed to investigate the effects of exosomal miR-133b derived from MSCs on glioma cell behaviors.

**Methods:**

Microarray-based analysis identified the differentially expressed genes (DEGs) in glioma. The expression patterns of EZH2 and miR-133b along with interaction between them were clarified in glioma. The expression of miR-133b and EZH2 in glioma cells was altered to examine their functions on cell activities. Furthermore, glioma cells were co-cultured with MSC-derived exosomes treated with miR-133b mimic or inhibitor, and EZH2-over-expressing vectors or shRNA against EZH2 to characterize their effect on proliferation, invasion, and migration of glioma cells in vitro. In vivo assays were also performed to validate the in vitro findings.

**Results:**

miR-133b was downregulated while EZH2 was upregulated in glioma tissues and cells. miR-133b was found to target and negatively regulate EZH2 expression. Moreover, EZH2 silencing resulted in inhibited glioma cell proliferation, invasion, and migration. Additionally, MSC-derived exosomes containing miR-133b repressed glioma cell proliferation, invasion, and migration by inhibiting EZH2 and the Wnt/β-catenin signaling pathway. Furthermore, in vivo experiments confirmed the tumor-suppressive effects of MSC-derived exosomal miR-133b on glioma development.

**Conclusion:**

Collectively, the obtained results suggested that MSC-derived exosomes carrying miR-133b could attenuate glioma development via disrupting the Wnt/β-catenin signaling pathway by inhibiting EZH2, which provides a potential treatment biomarker for glioma.

## Background

Gliomas are the most widespread and malignant types of tumors affecting the primary central nervous system [1]. Unfortunately, the incidence of gliomas ranges from 4.67 to 5.73 per 100,000 people [2]. Medical therapies for gliomas have made vast strides, but the mean survival of malignant glioma remains to be less than 1.5 years [3]. Despite advances in modern surgery and therapeutic strategies, such as chemotherapy and radiotherapy, the prognosis of glioma continues to be poor [4]. Interestingly, the role of mesenchymal stem cells (MSCs) in cancer progression has attracted a great deal of attention in recent times [5]. In addition, human mesenchymal stem cells (hMSCs) from adult bone marrow are known to be induced and differentiated into multiple mesenchymal tissues [6]. Moreover, researches have also shown that MSCs could alleviate glioma [7]. Therefore, our research interests arouse considering the possible mechanism of MSCs in glioma.

Additionally, stem cells possess the ability to release exosomes. The stem cell-derived exosomes function as a potential biomarker for the paracrine actions of MSCs [8]. Exosomes are already regarded as attractive targets for cancer treatment due to their extremely small sizes (40~100 nm) and great impact on affected cells [9]. The exact effects of exosomes on promotion or inhibition of glioma were previously investigated [10]. Exosomes, which encompass proteins, messenger RNAs (mRNAs), and microRNAs (miRNAs), can be transferred between cells [11]. MiRNAs are small non-coding RNA molecules which could regulate gene expressions at posttranscriptional levels by degrading or repressing their target miRNAs [12]. In addition, the effects of miRNAs on glioma have demonstrated increasing clinical implications [13]. Moreover, miR-133b has been documented to serve as a tumor suppressor in several cancers [14]. Similarly, miR-133b was further also reported to play an inhibitory role in glioblastoma [15]. Li et al. have also demonstrated the involvement of miR-133b in the growth and metastasis of glioma via regulation of the Sirt1 expression [16]. Furthermore, bioinformatics analysis predicted that miR-133b could target the Enhancer of Zeste 2 (EZH2) gene in glioma. EZH2 is a highly conserved gene in organisms and highly expressed in multiple human cancers [17]. EZH2 is also aberrantly expressed in glioma and exerts great effects on the invasive and metastatic abilities of glioblastoma [18]. In addition, downregulation of EZH2 has inhibitory impacts on glioma growth via suppression of the β-catenin signaling pathway [19]. The Wnt/β-catenin signaling pathway participates in the development of the central nervous system and is associated with oncogenesis in several tumors [20]. Wnt/β-catenin signaling pathway is implicated in glioblastoma progression [21]. However, the effects of MSC-derived exosomes on gliomas and the underlying mechanisms behind exosomal communication affecting tumor cells remain to be unclear. Therefore, we aim to explore the roles of exosomal communication in glioma microenvironment. According to the abovementioned evidence, we hypothesize that MSC-derived exosomes carrying miR-133b may provide therapeutic value for glioma treatment by regulating EZH2 and the Wnt/β-catenin signaling pathway.

## Materials and methods

### Ethics statement

The current study was approved by the Ethics Committee of the First Hospital of Jilin University. Signed informed consents and required documentation were obtained from each patient and respective guardian prior to the study. All procedures of animal experiments were in line with the Guide for Care and Use of Laboratory Animals published by the National Institutes of Health, and all efforts were made to minimize the suffering of the included animals.

### Study subjects

A total of 12 normal brain tissues and 54 glioma brain tissues were collected between January 2016 and September 2018 from the First Hospital of Jilin University. According to the 2016 World Health Organization (WHO) staging standard, the samples included 23 cases of glioma specimens at stage II, 18 cases of glioma specimens at stage III, and 13 cases of glioma specimens at stage IV (glioblastoma). None of the patients underwent radiotherapy prior to specimen collection. The obtained tissues were preserved in liquid nitrogen for the subsequent experiments.

Human normal brain glial cell line HEB and human glioma cell lines (U87, U251, LN229, and A172) were purchased from the Shanghai Institute of Biochemistry and Cell Biology (Shanghai, China). The aforementioned cells were incubated with Dulbecco’s modified Eagle medium (DMEM) (Gibco, NY, USA) supplemented with 10% fetal bovine serum (FBS) and penicillin-streptomycin (Gibco, NY, USA), respectively, and incubated with 5% CO_2_ in air at 37 °C. Next, the cells were detached with 0.25% trypsin and passaged (1: 3) and seeded into a 6-well plate (3 × 10^5^ cells/well). When cell confluence reached 70–80%, the cells at the logarithmic growth phase were selected for subsequent experimentation.

### Immunohistochemistry

Paraffin-embedded sections of normal brain tissues and glioma brain tissues were incubated with the primary antibody, rabbit anti-human EZH2 (dilution ratio of 1: 200, ab84989, Abcam Inc., Cambridge, MA, USA) at 4 °C overnight, and then with the goat anti-rabbit immunoglobulin G (IgG) (ab6785, dilution ratio of 1: 1000, Abcam Inc., Cambridge, MA, USA) secondary antibody. Subsequently, the samples were visualized using diaminobenzidine (DAB) (ST033, Guangzhou Weijia Technology Co., Ltd., Guangzhou, China), washed, and counterstained with hematoxylin (PT001, Shanghai Bogoo Biotechnology Co., Ltd., Shanghai, China) for 1 min. The sections were observed and photographed under a microscope.

### RNA isolation and quantitation

Total RNA content was extracted from fresh tissues and cells using Trizol kits (Invitrogen Inc., Carlsbad, CA, USA), and the total RNA (1 μg) was reverse transcribed into cDNA using the PrimeScript™ RT reagent with gDNA Eraser kits (RRO37A, Takara Bio Inc., Otsu, Shiga, Japan). RT-qPCR was conducted using the SYBR®*Premix Ex* Taq™ (Tli RNaseH Plus) kit (RR820A, Takara Bio Inc., Otsu, Shiga, Japan) and analyzed with the ABI7500 quantitative PCR instrument (Thermo Fisher Scientific Inc., Waltham, MA, USA). The system included SYBR® Premix Ex TaqTM II (10 uL), forward primer (0.8 uL), reverse primer (0.8 μL), ROX Reference Dye II (0.4 μL), cDNA (2 μL), and RNase Free ddH_2_O (6 μL). U6 served as the internal reference for miR-133b, and glyceraldehyde-3-phosphate dehydrogenase (GAPDH) was the internal reference of EZH2. The mRNA level patterns of the target gene were analyzed using the 2^−ΔΔCt^ method [22]. The primer sequences were provided by the Shanghai GenePharma Co. Ltd. (Shanghai, China) (Table 1).

**Table 1:** Primer sequences of the genes for RT-qPCR

Genes	Sequences
miR-133b	F: 5’-TTTGGTCCCCTTCAACCAGC-3’
	R: 5’-GTGCAGGGTCCGAGGT-3’
EZH2	F: 5′-GTGGAGAGATTATTCTCAAGATG-3′
	R: 5′-CCGACATACTTCAGGGCATCAGCC-3′
U6	F: 5′-CTCGCTTCGGCAGCACA-3’
	R: 5′-AACGCTTCACGAATTTGCGT-3’
GAPDH	F: 5′-GTCAACGGATTTGGTCTGTATT-3’
	R: 5′-CGCUUCACGAAUUUGCGUGUCAU-3’

*RT-qPCR* reverse transcription quantitative polymerase chain reaction, *miR-133b* microRNA-133b, *GAPDH* glyceraldehyde-3-phosphate dehydrogenase, *F* forward, *R* reverse

### Western blot analysis

The tissues were added with phenylmethylsulfonyl fluoride (PMSF) and protease inhibitors to extract the total protein content. The supernatant was extracted by centrifugation for 15 min at 40,256×*g* after pyrolysis at 4 °C. The protein concentration of each sample was determined using bicinchoninic acid (BCA) kits (23227, Thermo, Fisher Scientific Inc., Waltham, MA, USA). The uploading volume of the sample was controlled at 20 μg. The protein samples were separated by sodium dodecyl sulfate-polyacrylamide gel electrophoresis and transferred onto a polyvinylidene fluoride membrane. After being blocked with 5% bovine serum albumin for 1 h, the membrane was incubated with the primary antibodies, EZH2 (dilution ratio of 1:1000, ab186006), Wnt1 (dilution ratio of 1:100, ab85060), p-GSK-3β (dilution ratio of 1:500, PL0303230, PLlabs, Canada), GSK-3β (dilution ratio of 1:1000, ab93926), β-catenin (dilution ratio of 1:4000, ab6302), CD63 (dilution ratio of 1:1000, ab216130), HSP70 (dilution ratio of 1:1000, ab2787), and GAPDH (dilution ratio of 1:5000, ab8245) at 4 °C overnight. All the aforementioned antibodies except p-GSK-3β were purchased from Abcam Inc. (Cambridge, MA, USA). Subsequently, the samples were incubated with the horseradish peroxidase (HRP)-labeled goat anti-rabbit IgG (dilution ratio of 1: 20,000, ab205718, Abcam Inc., Cambridge, MA, USA) at 37 °C for 1.5 h. The samples were visualized using developer (NCI4106, Pierce, Rockford, IL, USA). The protein quantitative analysis, represented by the ratio of gray value between proteins and the internal reference (GAPDH), was conducted using the ImageJ 1.48u (Bio-Rad, Hercules, CA, USA).

### Cell treatment

Glioma U87 cells at the logarithmic growth phase were seeded into a 6-well plate at a density of 4 × 10^5^ cells/well. Upon reaching 70–80% confluence, the cells were treated with mimic-negative control (NC), miR-133b mimic, inhibitor-NC, miR-133b inhibitor, over-expression (oe)-NC, oe-EZH2, shRNA (sh)-NC, sh-EZH2, and miR-133b inhibitor + sh-EZH2 plasmids (10 μg,) according to the instructions of lipofectamine 2000 (11668-019, Invitrogen, New York, CA, USA) (10 μg per plasmid, and the final concentration was 50 nM). The transfection sequences and plasmids were purchased from Shanghai GenePharma Co. Ltd. (Shanghai, China).

### MSC isolation and characterization

The well-grown C57BL/6 mice were euthanized. Bone marrow cells of femur and tibia were suspended with DMEM complete medium containing 10% FBS (Biowest, Nuaillé, France) and penicillin-streptomycin (100 U/mL, Gibco Life Technologies, Grand Island, NY, USA). Subsequently, the cells were cultured at 37 °C with 5% CO_2_ in air. The medium was renewed after 3 days. The cells that did not adhere to the well were removed. Cell morphological changes were observed, photographed, and recorded in detail. Upon reaching 80–90% confluence, the cells were sub-cultured and collected for use when the cells reached at the third passage.

The MSCs at the third passage was re-suspended and adjusted to a concentration of 1 × 10^6^ cells/mL (200 μL) by PBS. The cell suspension of MSCs was incubated with 5 μL monoclonal antibodies labeled by different fluorescences (CD29, CD44, CD45, CD90, and Vimentin) at 4 °C avoiding exposure to light for 15 min. The fluorescence-labeled IgG antibody of the same color was applied as the isotypic control. The cells were then assessed using flow cytometry.

### Characterization of osteogenic/adipogenic induction

MSCs at the third passage were trypsinized and prepared into a cell suspension at a concentration of 1 × 10^5^ cells/mL, which was seeded in a 6-well plate. When the cells reached 60–70% confluence, the supernatant was discarded. The experimental group was treated with 2 mL of osteogenic induction complete medium (MUBMX-90021, Cyagen Biosciences Inc., Guangzhou, Guangdong, China), and the control group was treated with same amounts of DMEM complete medium. Thereafter, the fresh culture medium was replaced every 3 days for the experimental and control groups. After 2–3 weeks of induction, the culture solution of the experimental and control groups were removed and rinsed twice with PBS. Next, the cells in each well were fixed with 4% neutral formaldehyde for 30 min. After two PBS rinses, 1.5 mL of alizarin red was added to each well for 5-min reaction. The staining solution was removed, and cells were rinsed again twice with PBS. Then, the cells were observed and photographed under a microscope.

MSCs at the third passage were trypsinized and prepared into a cell suspension at a concentration of 3 × 10^5^ cells/mL, which was seeded in a 6-well plate. When the cells reached 80–90% confluence, the supernatant was discarded. The cells were treated with 2 mL of adipogenic induction medium A (MUBMX-90031, Cyagen Biosciences Inc., Guangzhou, Guangdong, China). Three days after induction, the liquid A in the 6-well plate was aspirated and 2 mL of the adipogenic induction medium B was added. After 24 h of induction, the liquid B was discarded and replaced with fresh adipogenic induction liquid A. After 5 alternate inductions, the cells were further induced for 7 days with the liquid B, during which the medium was renewed every 3 days. After induction, the liquid B in the 6-well plate was aspirated and rinsed twice with PBS. Then, the cells were fixed with 4% neutral formaldehyde for 30 min. After that, the formaldehyde was discarded. After two PBS rinses, 1.5 mL of Oil Red O dye was added to each well for 30-min reaction. The staining solution was removed, and the cells were rinsed again twice with PBS. Then, cells were observed and photographed under a microscope.

Alcian blue staining experiment was carried out with the kit provided by Guangzhou Exon Biotechnology Co., Ltd. (No. S-1504, Guangzhou, China). In order to induce chondrogenic differentiation, MSCs were collected in a 15-mL centrifugal tube and cultured in chondrogenic differentiation medium (Gibco BRL, Grand Island, NY, USA) at a rate of 2 × 10^5^ cells per tube. The medium was renewed every 3 days. After 21 days of chondrogenic differentiation, chondroid balls were prepared, and the MSCs were rinsed with PBS and fixed with 4% paraformaldehyde. The MSCs were rinsed by PBS twice, 2 min each time. Then, the MSCs were stained at room temperature for 30 min with 1% Alcian blue (pH 2.5). Subsequently, the MSCs were rinsed under running water for 2 min, soaked in deionized water twice, 2 min each time. Finally, the MSCs were observed under a microscope [23].

### Isolation of MSC-derived exosomal miR-133b

The exosomes in serum containing medium were removed by ultra-centrifugation at 100,000×*g* at 4 °C overnight. MSCs at the third passage or MSCs treated with different plasmids were incubated in DMEM overnight. When cell confluence reached about 80–90%, the MSCs were further incubated in exosome-free serum medium for 24 h and the supernatant was then collected. The cells were centrifuged at 2000*g* for 20 min at 4 °C to remove the cell debris, and the obtained supernatant was centrifuged at 10,000*g* for 1 h at 4 °C. The pellets were then suspended in serum-free DMEM containing 25 mM HEPES (pH = 7.4), and the previous high-speed centrifugation was repeated. The supernatant was discarded, and the pellets were stored at − 80 °C for later use [24]. The exosomes were then observed and photographed using transmission electron microscopy [25].

### Flow cytometry for identification of exosomes

The content of CD63, a surface marker of exosome, was detected by flow cytometry. The exosomes were resuspended by adding 1 mL PBS (containing 1% BSA), followed by incubation for 30 min at room temperature to block the non-specific antigen. The extracted exosomes from MSCs were incubated with the CD63-PE antibody for 30 min. The extracted exosomes from MSCs without treatment were regarded as the blank control and the extracted exosomes from MSCs incubated with PE-labeled IgG antibody served as the isotypic control. The Guava easyCyte™ system flow cytometer was applied for the detection. The miR-133b expression in exosomes was measured by qRT-PCR.

### Fluorescent labeling and transfer of exosomes

The extracted exosomes from MSCs were labeled with PKH26 (Red) (MINI67-1KT, Sigma-Aldrich Chemical Company, St Louis, MO, USA). Next, the fluorescent-labeled exosome was co-cultured with U87 cells for 48 h. The U87 cells were stained with 4′,6-diamidino-2-phenylindole (DAPI) (D4054, Yuheng Biotechnology Co., Ltd., Suzhou, Jiangsu, China) and observed under an inverted fluorescence microscope to determine whether U87 cells could endocytose the exosome from MSCs.

### Co-culture of exosomes and MSCs with glioma cells

U87 cells were plated in the basolateral chamber of a 24-well Transwell chamber (1 × 10^4^ cells/well), and the MSCs were plated in the apical chamber, which was co-cultured for 24 h. The U87 cells in the basolateral chamber were isolated using trypsinization to detect the expression of miR-133b and EZH2. In order to over-express or inhibit miR-133b in MSCs, miR-133b mimic and miR-133b inhibitor were used to treat the MSCs. Subsequently, the MSCs were treated with exosome inhibitor GW4869 to suppress the release of exosomes from MSCs and treated with 0.005% dimethylsulfoxide (DMSO) as control to observe whether the secretion of exosomes from MSCs treated with exosome inhibitor GW4869 was completely blocked under electron microscopy. MSCs at the logarithmic growth phase (2 × 10^5^ cells per well) were inoculated in a 6-well plate. When the cell confluence was 70–80%, the MSCs were transfected according to the lipofectamine 3000 instructions (L3000001, Invitrogen, New York, CA, USA). Next, the MSCs were treated with mimic-NC, miR-133b mimic, inhibitor-NC, or miR-133b inhibitor. Transfection sequence and plasmid were purchased from GenePharma (Shanghai, China).

The fluorescence-labeled exosomes from MSCs were treated with miR-133b mimic and miR-133b inhibitor, respectively, followed by co-culture with U87 cells over-expressing EZH2 with 50–60% confluence inoculated in 24-well plates for 48 h. The miR-133b expression and EZH2 mRNA level were determined using RT-qPCR, followed by detection of EZH2 protein level using Western blot analysis.

### 5-Ethynyl-2′-deoxyuridine (EdU) assay

EdU staining was conducted according to guidelines based on existing literature [26]. U87 cells were isolated from the co-culture system and inoculated in a 96-well plate (5 × 10^3^ cells/well). After incubation for 6 h, the cells were added with EdU medium (100 μL/well) for 2 h and cultured with 1 × Hoechst 33342 reaction solution (100 μL/well) avoiding exposure to light for 30 min. The photos were taken under a fluorescence microscope, and the number of EdU-labeled cells was recorded. The red-stained nuclei were regarded as positively labeled cells, and three fields were randomly selected to count the number of positive and negative cells under a microscope.

### Transwell assay

Transwell chambers (8 mm, Corning Glass Works, Corning, NY, USA) with polycarbonate film, with matrigel (for invasion experiment) and without matrigel (for migration experiment) were applied for cell invasion and migration detection on the 24-well plate. The basolateral chamber was added with 600 mL DMEM containing 20% FBS, and the apical chamber was added with U87 cells transfected for 48 h at a density of 1 × 10^6^ cells/mL. After incubation with 5% CO_2_ in air at 37 °C for 24 h, the samples were stained with 0.1% crystal violet for 5 min. A total of 5 fields were selected, and the cells were observed under an inverted microscope (TE2000, Nikon Corporation, Tokyo, Japan). The average number of cells was regarded as the number of cells through the chamber.

### Dual-luciferase reporter gene assay

The dual luciferase reporter gene vectors of EZH2 3′UTR and mutant plasmid with the miR-133b binding site, pmirGLO-EZH2-WT, and pmirGLO-EZH2-MUT were constructed. Subsequently, the reporter plasmid miR-133b mimic and NC plasmids were co-transfected into 293T cells. After transfection for 24 h, cell supernatant was collected. A Dual-Luciferase® Reporter Assay System (E1910, Promega, WI, USA) was employed to detect the luciferase activity in the samples. Each cell sample was added with 100 μL firefly luciferase working fluid to detect the firefly luciferase, and added with 100 μL renilla luciferase working fluid to detect the renilla luciferase. The ratio of firefly luciferase and renilla luciferase represented the relative luciferase activity.

### Tumorigenicity assay in nude mice

A total of 20 immunodeficient female mice (aged 3–5 weeks old, weighing 18–21 g) with the specific pathogen-free (SPF) grade were purchased from Shanghai Laboratory Animal Center of Chinese Academy of Science (Shanghai, China). The cultured U87 cells were suspended with PBS at a concentration of 1 × 10^6^ cells/mL. The cell suspension (50 μL) was subcutaneously injected into the right hindlimb of the nude mice [27]. On the 5th, 10th, 15th, 20th, and 25th days, nude mice were injected with normal saline or exosomes via the tail vein at a dose of 200 μL and a concentration of 1 μg/μL. After 30 days, the nude mice were euthanized. The tumor volume (> 500 mm^3^) and weight were measured and recorded. Peripheral blood samples were collected once the mice were euthanized to prepare serum. The exosomes were extracted from the serum using the abovementioned method, and RT-qPCR was employed to determine miR-133b expression. The tumor tissues were fixed in 10% formaldehyde, followed by dehydration, paraffin-embedding, and cutting into 4-μm sections for subsequent experimentation. The protein levels of Wnt1, p-GSK-3β, GSK-3β, and β-catenin were assessed using Western blot analysis.

### Statistical analysis

Statistical analyses were performed using the SPSS 21.0 software (IBM Corp. Armonk, NY, USA). Measurement data were shown as mean ± standard deviation. Comparisons between two groups were analyzed using the unpaired *t* test, while comparisons among multiple groups were analyzed using one-way analysis of variance (ANOVA) with Dunnett’s post hoc test. Data analyses at different time points were carried out by repeated measures ANOVA with Bonferroni post hoc test. A value of *p* < 0.05 was considered to be statistically significant.

## Results

### EZH2 is upregulated in glioma

**Fig. 1:**
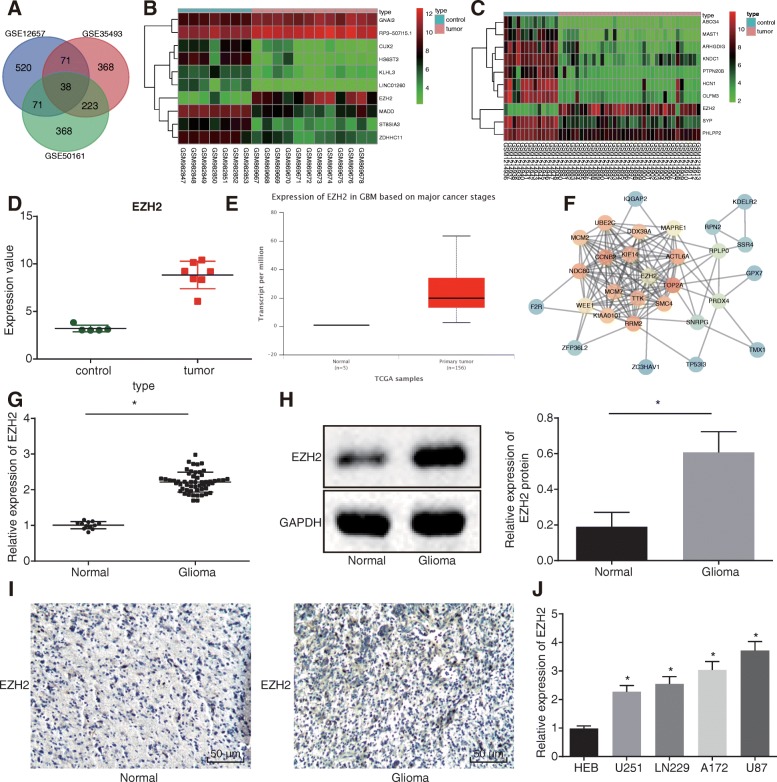
EZH2 is highly expressed in glioma tissues and cells. **a** The intersection of the top 700 DEGs in GSE12657, GSE35493, and GSE50161. **b**, **c** Heat maps of DEGs in GSE35493 and GSE50161. **d** EZH2 gene expression profile analysis in GSE12657; green represents the gene expression of normal samples, and red represents the gene expression of glioma samples. **e** EZH2 gene expression determined in normal and glioma samples based on the TCGA database. **f** Gene network map of EZH2 and its related genes generated using Cytospace software. **g** EZH2 mRNA level measured in normal brain tissues (*n* = 12) and glioma tissues (*n* = 54) using RT-qPCR. **h** EZH2 protein level measured in normal brain tissues (*n* = 12) and glioma tissues (*n* = 54) using Western blot analysis. **i** Positive expression of EZH2 detected in glioma using immunohistochemistry (200×). **j** EZH2 expression measured in human normal brain glial cell line HEB and human glioma cell lines (U87, U251, LN229, and A172) using RT-qPCR; *p < 0.05 vs. normal brain tissues/HEB cell line; measurement data were shown as mean ± standard error; comparisons between two groups were conducted by unpaired *t* test, and comparisons among multiple groups were assessed by one-way analysis of variance (Dunnett’s post hoc test). The experiment was repeated three times to obtain the mean value

Initially, we retrieved the microarray datasets GSE12657 (5 cases of normal samples and 7 cases of glioma samples), GSE35493 (7 cases of normal samples and 12 cases of glioma samples), and GSE50161 (13 cases of normal samples and 34 cases of glioma samples) from the GEO database. Subsequently, after analyzing the DEGs in glioma samples and normal control samples, a total of 1941, 4888, and 4935 DEGs were obtained in the three respective microarray datasets. Afterwards, Venn analyses were performed on the top 1000 DEGs in GSE12657, GSE35493, and GSE50161 to obtain the intersection of DEGs in the three microarray datasets (Fig. 1a), which revealed 38 DEGs. Among them, EZH2 presented as the most significantly upregulated DEG (logFC > 1). GSE35493 and GSE50161 were Matrix thermography of DEGs (Fig. 1b, c). Based on the GSE12657 dataset, GraphPad Prism 6 was applied to generate the EZH2 gene expression profile, which revealed that EZH2 was highly expressed in glioma samples (Fig. 1d). In addition, the TCGA database also demonstrated that EZH2 was highly expressed in glioma samples (Fig. 1e). Next, the String website (https://string-db.org/) was used to search the relationship among the genes, while the Cytospace software was applied to generate the gene network map. The EZH2 gene was found to be at the center of the gene network, and there were relationships among the EZH2 gene and other DEGs (Fig. 1f). Moreover, a recent study also demonstrated that EZH2 is related to the development of glioma and affects glioma cell invasion and metastasis [28]. In addition, the EZH2 gene presents with abnormally high expression in glioma, which is a key regulator of invasion and metastasis [18].

RT-qPCR and Western blot analysis revealed increased EZH2 expression in brain tissues of patients with glioma (*p* < 0.05) (Fig. 1g, h). Immunohistochemistry demonstrated that the positive expression of EZH2 in glioma tissues was located in the nucleus, presented as brownish-yellow coloration, while the positive expression rate of EZH2 in glioma tissues was elevated (*p* < 0.05) (Fig. 1i). Moreover, compared with HEB cells, EZH2 was found to be highly expressed in U87, U251, LN229, and A172 cell lines, and EZH2 was the highest in the U87 cells (*p* < 0.055) (Fig. 1j). Overall, elevated EZH2 expression was identified in glioma tissues and cells.

### Silencing of EZH2 inhibits cell proliferation, migration, and invasion in glioma U87 cells

**Fig. 2:**
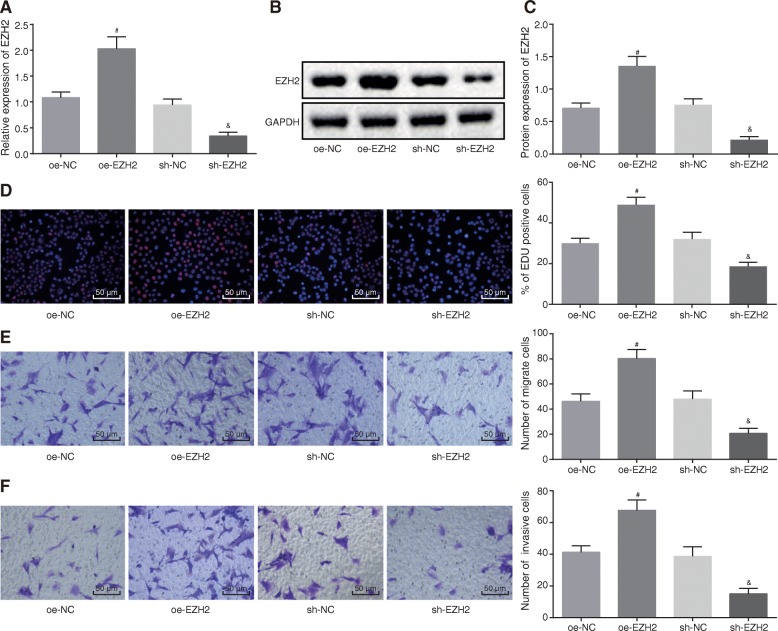
Silencing of EZH2 suppresses cell proliferation, invasion, and migration in glioma. **a** EZH2 mRNA level determined in glioma cells treated with oe-EZH2 or sh-EZH2 using RT-qPCR. **b** EZH2 protein band patterns detected in glioma cells treated with oe-EZH2 or sh-EZH2. **c** EZH2 protein level measured in glioma cells treated with oe-EZH2 or sh-EZH2 using Western blot analysis. **d** Proliferation of glioma cells treated with oe-EZH2 or sh-EZH2 detected using EdU assay (200×). **e** Migration of glioma cells treated with oe-EZH2 or sh-EZH2 detected using Transwell assay (200×). **f** Invasion of glioma cells treated with oe-EZH2 or sh-EZH2 detected using Transwell assay (200×); ^#^*p* < 0.05 vs. glioma cells treated with oe-NC; ^&^*p* < 0.05 vs. glioma cells treated with sh-NC; measurement data were shown as mean ± standard error; comparisons between two groups were conducted by unpaired *t* test. The experiment was repeated three times to obtain the mean value

After altering the expression of EZH2 in U87 cells, we employed RT-qPCR and Western blot analysis (Fig. 2a–c), which showed increased EZH2 expression in glioma cells treated with oe-EZH2, whereas decreased EZH2 expression was noted in U87 cells treated with sh-EZH2 (*p* < 0.05). EdU assay and Transwell assay revealed that oe-EZH2 treatment promoted proliferation of EdU-positive cells along with the U87 cell migration and invasion, while U87 cells treated with sh-EZH2 inhibited the EdU-positive cell proliferation, as well as cell migration and invasion (*p* < 0.05) (Fig. 2d–f). These findings demonstrated that silencing of EZH2 could suppress cell proliferation, invasion, and migration in glioma.

### miR-133b targets and negatively regulates EZH2

**Fig. 3:**
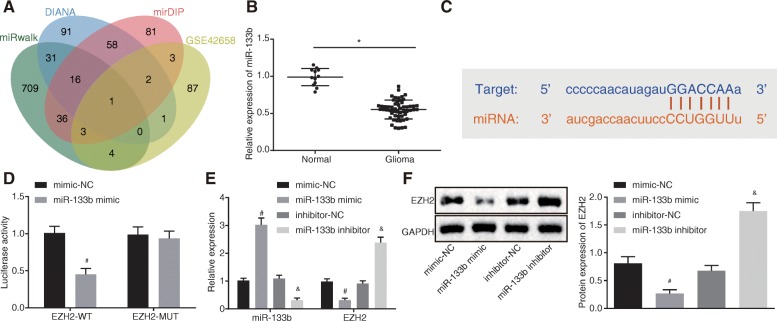
miR-133b targets and negatively regulates EZH2. **a** Venn analysis of miRNAs that regulate EZH2; blue represents the predicted results of DIANA database, red represents the predicted results of mirDIP database, green represents the predicted results of mirWalk database, and yellow represents the differentially expressed downregulated miRNAs in GSE42658. **b** miR-133b expression in normal brain tissues (*n* = 12) and glioma tissues (*n* = 54) determined by RT-qPCR. **c** Binding site prediction of miR-133b in EZH2 3′UTR. **d** Detection of luciferase activity using dual-luciferase reporter gene assay. **e** miR-133b expression and EZH2 mRNA level after miR-133b over-expression and inhibition using RT-qPCR. **f** EZH2 protein level after miR-133b over-expression and inhibition using Western blot analysis; *p < 0.05 vs. normal brain tissues; ^#^*p* < 0.05 vs. glioma cells treated with mimic-NC; ^&^*p* < 0.05 vs. glioma cells treated with inhibitor-NC; measurement data were shown as mean ± standard deviation; comparisons between two groups were conducted by unpaired *t* test, and comparisons among multiple groups were assessed by one-way analysis of variance (Dunnett’s post hoc test). The experiment was repeated three times to obtain the mean value

Following verification of over-expression of EZH2 in glioma cells, we further explored the miRNAs that regulate the EZH2 gene. Bioinformatics websites, mirWalk database, (http://mirwalk.umm.uni-heidelberg.de/), DIANA database (http://diana.imis.athena-innovation.gr/DianaTools/index.php?r=microT_CDS/index), and mirDIP database (http://ophid.utoronto.ca/mirDIP/), combined with Venn mapping, were employed to predict the miRNAs that regulate EZH2. The top 750 miRNAs were selected in the current study, and only one miRNA appeared at the intersection of 750 miRNAs and differentially expressed miRNAs (logFC > 1) in GSE42658, specifically, miR-133b (Fig. 3a). Next, RT-qPCR (Fig. 3b) revealed that miR-133b was downregulated in glioma. Online analysis software revealed the existence of a specific binding region between EZH2 and miR-133b (Fig. 3c). Subsequent dual-luciferase reporter gene assay verified that EZH2 was indeed a target gene of miR-133b. The luciferase activity of WT EZH2 3′-UTR was suppressed by miR-133b (*p* < 0.05), while no differences were observed in MUT EZH2 3′-UTR (*p* > 0.05) (Fig. 3d), suggesting that miR-133b could specifically bind to EZH2. Furthermore, we altered the expression of miR-133b in U87 cells, and RT-qPCR and Western blot analysis (Fig. 3e, f) demonstrated decreased EZH2 mRNA and protein levels in cells treated with miR-133b mimic, while opposite trends were observed in cells treated with miR-133b inhibitor (*p* < 0.05). Therefore, the obtained data suggested that EZH2 was targeted and negatively regulated by miR-133b.

### Characterization of MSCs and MSC-secreted exosomes

**Fig. 4:**
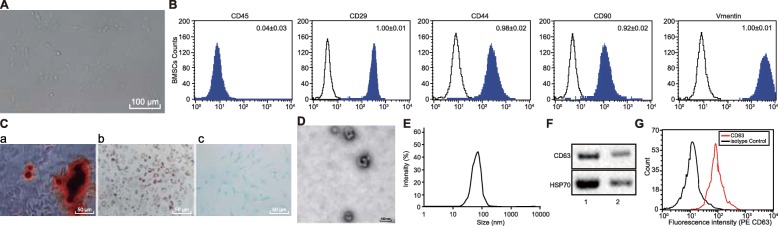
Characterization of MSCs and MSC-secreted exosomes. **a** Morphological observation of MSCs (100×). **b** Identification of surface marker molecules in MSCs using flow cytometry. **c** Osteogenic and adipogenic induction culture of MSCs (400×). (a) Osteogenic differentiation evaluated by alizarin red staining; (b) adipogenic differentiation evaluated by oil red O staining; (c) chondral differentiation-Alcian blue staining (200×). **d** Identification of exosomes by a transmission electron microscopy. **e** Detection of exosome diameter by dynamic light scattering. **f** Exosome surface markers CD63 and HSP70 measured using Western blot analysis; lane 1, extracted exosomes; lane 2, supernatant after extraction of exosomes. **g** The content of CD63, a surface marker in exosomes, detected using flow cytometry; *p < 0.05 vs. lane 1; measurement data were shown as mean ± standard error. The experiment was repeated three times to obtain the mean value

After uncovering the binding between miR-133b and EZH2, we shifted our focus in identifying the influence of MSCs and exosomes from MSCs on glioma cells. In addition, it has been previously suggested that MSCs could help attenuate the development of glioma [7]. Thus, the current study investigated the protective roles of MSCs and exosomes in glioma. Firstly, the MSCs were isolated. After 3 days of seeding, MSCs began to adhere to the wells. Morphologically, the MSCs were spindle-shaped. The cells grew in a whirlpool or cluster with clear nuclei, showing typical cell characteristics of MSCs (Fig. 4A). Flow cytometry displayed high expression of CD90, CD29, and CD44, along with low CD45 expression in the isolated MSCs, which were consistent with the biological characteristics of MSCs (Fig. 4B). The observation of osteogenesis of MSCs following alizarin red staining demonstrated that, after 21 days of osteogenic induction, the cells overlapped in layers and formed calcified nodules containing a small amount of mineral salt deposition, indicating that the cells have the potential to differentiate into osteoblasts (Fig. 4C (a)). After 25 days of adipogenic induction, lipid deposits were observed in the cells, and lipid droplets became larger or beaded, highlighting the potential to differentiate into adipocyte (Fig. 4C (b)). On the 21st day of chondrogenesis, the presence of glycosaminoglycan was demonstrated by Alcian blue staining, which indicated that glycosaminoglycan had the potential to differentiate into chondrogenesis (Fig. 4C (c)).

Under transmission electron microscopy, the exosomes presented with similar morphology as round or oval membranous vesicles (Fig. 4D). Dynamic light scattering detection demonstrated exosome particles with diameters ranging from 30 to 120 nm (Fig. 4E). Western blot analysis revealed that CD63 and HSP70 were upregulated in exosomes (Fig. 4F), and flow cytometry illustrated that the content of CD63, a surface marker of exosome, was significantly elevated in exosomes (*p* < 0.05) (Fig. 4G).

### MSC-derived exosomal miR-133b inhibits EZH2 expression in glioma U87 cells

**Fig. 5:**
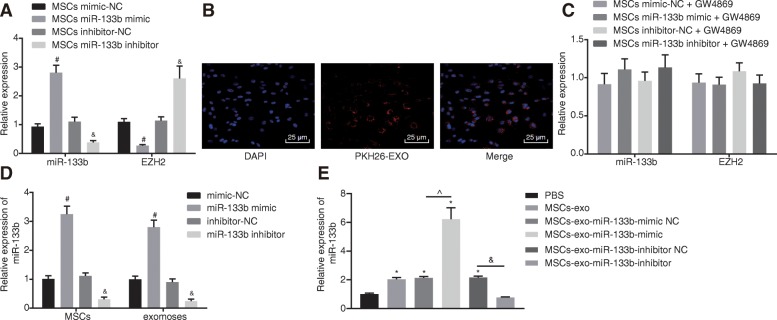
MSCs transfer miR-133b to glioma cells through exosomes to repress the expression of EZH2. **a** Expression of miR-133b and EZH2 in MSCs co-cultured with glioma cells after restoration or depletion of miR-133b determine using RT-qPCR. **b** Internalization of exosomes by glioma cells observed under the inverted microscope (400×). **c** miR-133b and EZH2 expression in MSCs treated with GW4869 co-cultured glioma cells after restoration or depletion of miR-133b measured using RT-qPCR. **d** miR-133b expression in exosomes and MSCs after restoration or depletion of miR-133b measured using RT-qPCR. **e** miR-133b expression in glioma cells co-cultured with MSC-derived exosome by RT-qPCR; ^#^*p* < 0.05 vs. MSCs co-cultured with glioma cells treated with mimic-NC; ^&^*p* < 0.05 vs. MSCs co-cultured with glioma cells treated with inhibitor-NC; *p < 0.05 vs. glioma cells co-cultured with PBS; ^^^*p* < 0.05 vs. glioma cells co-cultured with miR-133b mimic NC; ^@^*p* < 0.05 vs. glioma cells co-cultured with miR-133b inhibitor NC; measurement data were shown as mean ± standard deviation; comparisons among multiple groups were assessed by one-way analysis of variance (Dunnett’s post hoc test). The experiment was repeated three times to obtain the mean value

After identifying MSCs and MSC-secreted exosomes, we further investigated the regulatory effect of MSC-derived exosomal miR-133b on EZH2. MSCs were co-cultured with U87 cells to determine the expression patterns of miR-133b and EZH2 in glioma cells using RT-qPCR. The findings demonstrated that MSCs co-cultured with glioma cells treated with miR-133b mimic exhibited decreased EZH2 expressions and increased miR-133b expression, while cells treated with miR-133b inhibitor displayed the opposite results (*p* < 0.05) (Fig. 5a). Thus, MSCs affected miR-133b and EZH2 expression in glioma cells.

After co-culture of exosomes with glioma cells for 48 h, confocal fluorescence microscopy was applied to observe the internalization of exosomes by glioma cells, which showed red fluorescence around glioma cells, indicating the internalization of PKH26-exosomes by glioma cells and that exosomes could be transferred from the MSCs of donor cells to recipient glioma cells (Fig. 5b). Next, MSCs were treated with the exosome inhibitor GW4869 to inhibit exosome secretion to investigate the role of exosomes in this process. MSCs from different groups treated with GW4869 were co-cultured with glioma cells. The expression of miR-133b and EZH2 in MSCs co-cultured glioma cells was detected using RT-qPCR, which showed that MSCs had no impact on miR-133b and EZH2 expression in U87 cells after the blockade of exosomes (Fig. 5c). Subsequently, MSCs were treated with miR-133b mimic or inhibitor to interfere with the miR-133b expression. After restoring or downregulating miR-133b, RT-qPCR revealed that miR-133b expression was elevated in MSCs and MSC-derived exosomes co-cultured glioma cells treated with miR-133b mimic, while opposite trends were observed in cells treated with miR-133b inhibitor (*p* < 0.05) (Fig. 5d). The exosomes treated with different plasmids were co-cultured with U87 cells, and the expression of miR-133b in U87 cells was detected by RT-qPCR (Fig. 5e). Compared with U87 cells co-cultured with PBS, miR-133b expression in U87 cells was significantly increased in U87 cells co-cultured with MSC-derived exosomes and U87 cells co-cultured with MSC-derived exosomes treated with miR-133b mimic, while being decreased in U87 cells co-cultured with MSC-derived exosomes treated with miR-133b inhibitor. The aforementioned results indicated that exosomes play a significant role in transferring exogenous miR-133b from MSCs to glioma cells.

### MSC-derived exosomal miR-133b inhibits cell proliferation, migration, and invasion in glioma U87 cells via inhibition of Wnt/β-catenin signaling pathway

**Fig. 6:**
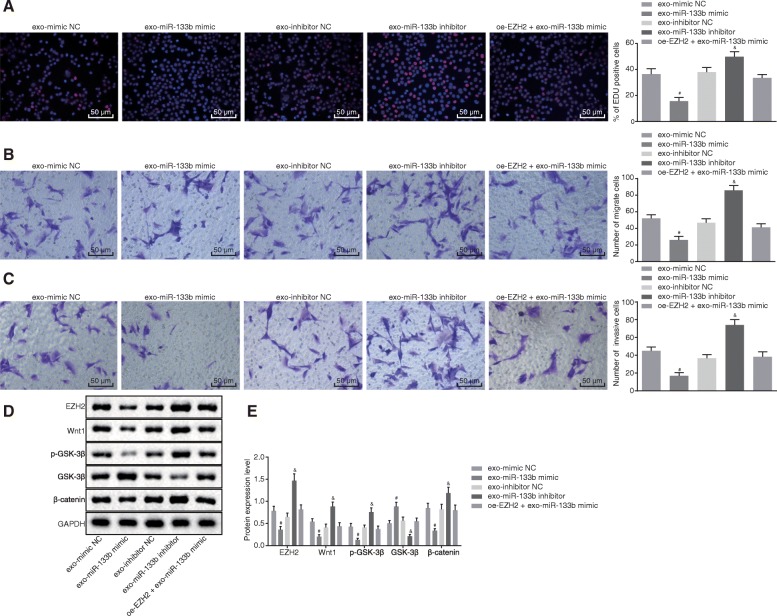
Exosomal miR-133b derived from MSCs represses glioma cell proliferation, migration, and invasion via inhibition of the Wnt/β-catenin signaling pathway. **a** Proliferation of glioma cells treated with exo-miR-133b mimic, exo-miR-133b inhibitor, or oe-EZH2 + exo-miR-133b mimic detected using EdU assay (200×). **b** Migration of glioma cells treated with exo-miR-133b mimic, exo-miR-133b inhibitor, or oe-EZH2 + exo-miR-133b mimic detected using Transwell assay (200×). **c** Invasion of glioma cells treated with exo-miR-133b mimic, exo-miR-133b inhibitor, or oe-EZH2 + exo-miR-133b mimic detected using Transwell assay (200×). **d** Protein band patterns of EZH2, Wnt1, p-GSK-3β, GSK-3β, and β-catenin detected in glioma cells treated with exo-miR-133b mimic, exo-miR-133b inhibitor, or oe-EZH2 + exo-miR-133b mimic. **e** Protein levels of EZH2, Wnt1, p-GSK-3β, GSK-3β, and β-catenin measured in glioma cells treated with exo-miR-133b mimic, exo-miR-133b inhibitor, or oe-EZH2 + exo-miR-133b mimic using Western blot analysis; ^#^*p* < 0.05 vs. glioma cells treated with exo-mimic NC; ^&^*p* < 0.05 vs. glioma cells treated with exo-inhibitor NC; measurement data were shown as mean ± standard deviation; comparisons among multiple groups were assessed by one-way analysis of variance (Dunnett’s post hoc test). The experiment was repeated three times to obtain the mean value

Additionally, exosomes derived from MSCs were co-cultured with glioma cells. EdU assay and Transwell assay revealed that cells treated with exo-miR-133b mimic exhibited proliferation of EdU-positive cells and U87 cell migration and invasion, while glioma cells treated with exo-miR-133b inhibitor displayed the opposite results (*p* < 0.05), and no obvious differences were detected in cells treated with oe-EZH2 + exo-miR-133b mimic (*p* > 0.05) (Fig. 6a–c).

We inferred that EZH2 may play a role through the Wnt/β-catenin signaling pathway. Western blot analysis revealed that cells treated with exo-miR-133b mimic showed reduced protein levels of Wnt1, p-GSK-3β, and β-catenin and increased levels of GSK-3, while the opposite results were observed in cells treated with exo-miR-133b inhibitor (*p* < 0.05), and no significant differences were found in cells treated with oe-EZH2 + exo-miR-133b mimic (*p* > 0.05) (Fig. 6d, e). Therefore, it was demonstrated that exosomal miR-133b derived from MSCs repressed cell proliferation, migration, and invasion in glioma by inhibiting the Wnt/β-catenin signaling pathway.

### MSC-derived exosomal miR-133b inhibits tumor growth of glioma in vivo

**Fig. 7:**
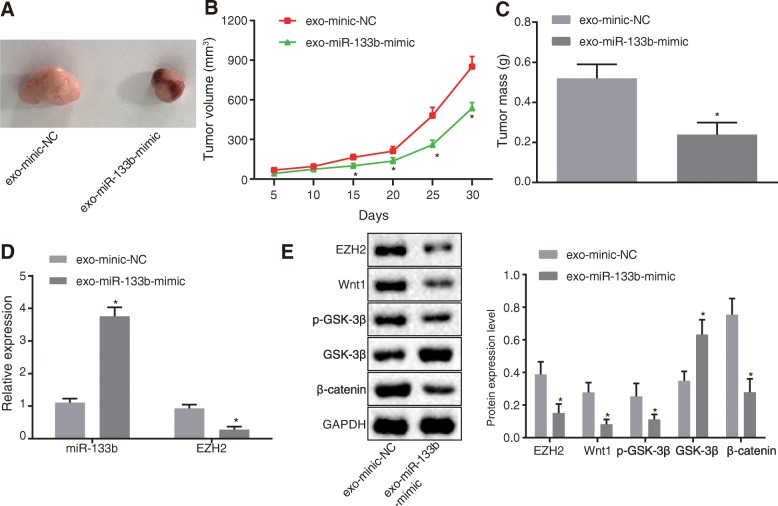
MSC-derived exosomal miR-133b suppressed tumor growth of glioma in vivo. **a** Tumorigenesis of nude mice (*n* = 6). **b** Tumor volume of nude mice (*n* = 6). **c** Tumor weight of nude mice (*n* = 6). **d** Expression of miR-133b and EZH2 determined using RT-qPCR. **e** Protein levels of EZH2, Wnt1, p-GSK-3β, GSK-3β, and β-catenin measured using Western blot analysis; ^#^*p* < 0.05 vs. nude mice injected with exo-mimic NC; ^&^*p* < 0.05 vs. glioma cells injected with exo-inhibitor NC; measurement data were shown as mean ± standard deviation; comparisons between two groups were conducted by unpaired *t* test; The data analysis at different time points was performed by repeated measures ANOVA with Bonferroni post hoc test. The experiment was repeated three times to obtain the mean value

Finally, nude mice were inoculated with U87 cells, and the tumorigenic rate was calculated to be 90%. After establishment of the glioma nude mice models, the mice were intraperitoneally injected with exosomes from MSCs with different treatments and euthanized after 30 days. The gliomas were dissected and presented with oval or irregular shapes. All tumors were confirmed by pathological examination. Compared with nude mice with glioma and mice injected with exo-mimic-NC, the mice injected with exo-miR-133b-mimic showed a reduction in tumorigenic ability, tumor volume, and weight (*p* < 0.05) (Fig. 7a–c).

RT-qPCR revealed elevated miR-133b expressions and decreased EZH2 mRNA levels in the tumor tissues of mice injected with exo-miR-133b-mimic relative to mice injected with exo-mimic-NC (*p* < 0.05) (Fig. 7d). Western blot analysis demonstrated reduced protein levels of EZH2, Wnt1, p-GSK-3β, and β-catenin and increased protein levels of GSK-3β (*p* < 0.05) (Fig. 7e). The aforementioned findings demonstrated that MSC-derived exosomal miR-133b inhibited the tumor growth of glioma in vivo*.*

## Discussion

Exosomes, nanoscale vesicles secreted by numerous cells, exert their involvement in intracellular communication and material transport by directing the action of signal molecules on the surface of the cell membrane and mediation of cell content during membrane fusion [29]. Remarkably, recent evidence has indicated that exosomes derived from MSCs could possibly serve as mediators in glioma development [30]. Therefore, the current study aimed to explore the effects of MSC-derived exosomes over-expressing miR-133b on the regulation of glioma cell activity. Taken together, our findings revealed that MSC-derived exosomes over-expressing miR-133b could inhibit the progression of glioma by targeting EZH2 through the Wnt/β-catenin signaling pathway.

Firstly, our results demonstrated that glioma tissues and cells exhibit downregulated expressions of miR-133b. Similarly, Brower et al. verified that several miRNAs, playing roles in tumorigenesis, often present with dysregulated expressions in malignant cells relative to normal cells [31]. Notably, miR-133b is well-documented to exhibit poorly expressions in a variety of cancers [14]. Of note, miR-133b expression obviously reduced in glioma tissues [16]. Additionally, the data in our study further indicated that miR-133b targeted the EZH2 gene, as evidenced by upregulated levels in glioma tissues and cells. Moreover, Orzan et al. illustrated that EZH2 is upregulated in malignant gliomas [17]. In addition, Wu et al. also documented elevated expressions of EZH2 in gliomas and proposed that EZH2 plays a regulatory role in glioma development [32]. These findings support that miR-133b is poorly expressed, while EZH2 is highly expressed in glioma, wherein miR-133b targets and negatively regulates the expression of EZH2.

Furthermore, our findings revealed that MSCs could upregulate and transfer miR-133b to glioma cells via the route of exosome cargo. Exosomal miRNAs are not only implicated in the interactions within the tumor microenvironment, but also mediate communication between the extrinsic environment and tumor microenvironment [33]. A previous study further proved that MSCs could potentially help curb the development of glioma [7]. Moreover, MSC-derived exosomes also possess the ability to transfer miRNAs so as to promote the regulatory function of miRNA in the progression of glioblastoma [10]. Overall, these discoveries emphasize that MSCs elevate and transfer miR-133b to glioma cells through exosomes, thereby enhancing the effects of miR-133b.

Additionally, the in vitro and in vivo experiments performed in our study revealed that MSC-derived exosomal miR-133b inhibited glioma cell proliferation, invasion, and migration and tumor growth by reducing EZH2 by inhibiting the Wnt/β-catenin signaling pathway. Another study depicted consistent findings suggesting that MSCs could transfer miRNAs to glioma cells to inhibit cell migration and tumor growth in glioma [34]. Furthermore, miR-133b has also been previously proven to suppress glioblastoma cell migration and invasion by targeting MMP14 [15]. Likewise, Li et al. implied that the restoration of miR-133b suppresses the proliferation and invasion of U87 cells as a result of Sirt1 downregulation [16]. Strikingly, EZH2 is also well-known to serve as an important regulator of cell invasion and metastasis in glioma [18]. In addition, another study reported that depletion of EZH2 could repress the proliferation of glioma cells, while we witnessed a similar function in the current study [17]. In line with our findings, EZH2 has been previously associated with the Wnt/β-catenin signaling pathway, and upregulation of EZH2 activated the Wnt/β-catenin signaling pathway to affect proliferation and migration in tumor cells [35, 36]. The EZH2/miR-328/β-catenin signaling cascade serves as a novel therapeutic biomarker for glioma, whereas inhibition of EZH2 is also associated with the suppression of glioma growth by restraining the β-catenin signaling pathway [19]. The Wnt/β-catenin signaling pathway is commonly dysregulated in the progression of several tumors, including glioma [21]. Moreover, a previous study demonstrated that suppression of Wnt/β-catenin reduced the proliferation, migration, and invasion of U87 glioma cells [20]. All in all, the aforementioned results illustrate that MSC-derived exosomes carrying miR-133b inhibit the progression of glioma via inhibition of EZH2 and the Wnt/β-catenin signaling pathway. Therefore, it is reasonable to propose that MSCs delivering miR-133b could offer potential a therapeutic value in future glioma treatment.

## Conclusion

**Fig. 8:**
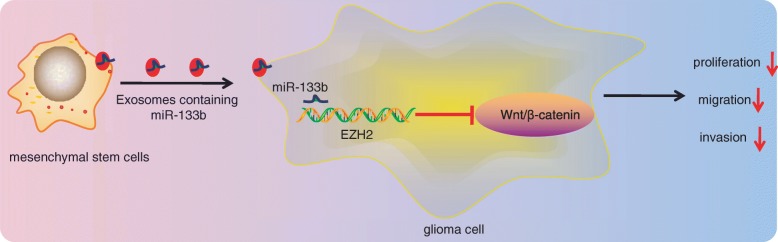
The schematic representation of mechanism by which MSC-derived exosomes containing miR-133b affect glioma cell activities. MSCs transfer miR-133b to glioma cells through exosomes to inhibit the EZH2 expression via suppression of the Wnt/β-catenin signaling pathway, whereby the glioma cell proliferation, migration, and invasion are diminished, and ultimately, the progression of glioma is attenuated

In conclusion, our finding evidenced that MSC-derived exosomes transferring miR-133b into glioma cells could potentially inhibit EZH2 expression via blocking the Wnt/β-catenin signaling pathway, thereby suppressing cell proliferation, migration, and invasion in glioma (Fig. 8). Thus, exosomes secreted from MSCs carrying upregulated miR-133b may function as a promising therapeutic biomarker for anti-cancer treatment of glioma. However, our research is still at the preclinical stage, and the investigations on the mechanism of action are still insufficient. Due to the limited understanding, future researches are warranted in order to further explore the intrinsic mechanisms highlighted in our study.

## Data Availability

The datasets generated/analyzed during the current study are available.

## Abbreviations

MSCs: Mesenchymal stem cells
miRNAs: MicroRNAs
DEGs: Differentially expressed genes
hMSCs: Human mesenchymal stem cells
mRNAs: Messenger RNAs
EZH2: Enhancer of Zeste 2
WHO: World Health Organization
IgG: Immunoglobulin G
GAPDH: Glyceraldehyde-3-phosphate dehydrogenase
PMSF: Phenylmethylsulfonyl fluoride
HRP: Horseradish peroxidase
DMEM: Dulbecco’s modified Eagle medium
FBS: Fetal bovine serum
DMSO: Dimethylsulfoxide
